# Editorial: Children's neurodevelopment in the post-COVID era: From hospital to community

**DOI:** 10.3389/fped.2022.976884

**Published:** 2022-10-12

**Authors:** Gary Diamond, Joav Merrick

**Affiliations:** ^1^ Alyn Hospital for Children's Rehabilitation, Jerusalem, Israel; ^2^ Clalit Health Services, Children's Developmental Services: Jaljulya-Kfar Kasem and Ramleh Centers, Tel Aviv, Israel; ^3^ National Institute of Child Health and Human Development, Jerusalem, Israel; ^4^ Department of Pediatrics, Mt Scopus Campus, Hadassah Medical Center and Faculty of Medicine, Hebrew University of Jerusalem, Jerusalem, Israel; ^5^ Kentucky Children’s Hospital, University of Kentucky, Lexington, KY, United States; ^6^ Center for Healthy Development, School of Public Health, Georgia State University, Atlanta, GA, United States

**Keywords:** early intervention, COVID 19, parental support, developmental teams, quarantine, pandemic, autis spectrum disorder, brain structural alterations

**Editorial on the Research Topic** Children's neurodevelopment in the post-COVID era: From hospital to community By Diamond G, Merrick J. (2022) Front. Pediatr. 10: 976884. doi: 10.3389/fped.2022.976884

## Introduction

Even in the best of times, caring for the developmental needs of children in general, and of the handicapped in particular, is no simple task. When society faces a monumental crisis and public services are challenged with on-going patient care and health delivery compromised, the task is all the more arduous. Such was the case of the recently abated Sars-CoV-2 pandemic (COVID-19), with its recurrent waves of mutated variants. With the last Omicron variant wave having abated, life and public services are slowly returning to normal, with its vestiges still plaguing the economy and the health system.

Neurological involvement in adults with varying degrees of severity of SARS-CoV-2 has been reported in the literature. There is strong evidence of brain-related abnormalities in COVID-19 among adults. Investigated brain changes in 785 participants of a UK Biobank (aged 51–81 years) who were imaged twice using magnetic resonance imaging, including 401 cases who tested positive for infection with SARS-CoV-2 between their two scans—with 141 days on average separating their diagnosis and the second scan—as well as 384 controls. Identified were significant longitudinal effects when comparing the two groups, including (1) a greater reduction in grey matter thickness and tissue contrast in the orbitofrontal cortex and parahippocampal gyrus; (2) greater changes in markers of tissue damage in regions that are functionally connected to the primary olfactory cortex and (3) a greater reduction in global brain size in the SARS-CoV-2 cases. The participants who were infected with SARS-CoV-2 also showed on average a greater cognitive decline between the two time points (1).

In a recently published exhaustive survey of the direct and indirect effects of Sars-CoV-2 on the neuro-developmental functioning of infants and children, Carl Stafstrom noted (2):

“The new coronavirus, SARS-CoV-2, can affect CNS and peripheral nervous system function in both children and adults, although symptoms are usually less severe at younger ages. Much more research is needed on the role of brain development in COVID-19 manifestations and outcomes. At any age, neurological involvement can be specific or non-specific and involve decreased taste/smell, headache, myalgia, confusion/encephalopathy, strokes, seizures, and a variety of other symptoms. The long-term consequences of both the virus infection itself and secondary effects of quarantine, isolation, family illness, and other stressors mandate that clinicians should monitor chronic symptoms and maintain a high degree of vigilance over time and in an age-specific manner. As the COVID-19 pandemic continues and evolves, continuing and new/emerging challenges face the healthcare system (2).”

“Data supporting any of the postulated (direct viral or inflammatory cascade mechanisms} in the CNS are scarce. Whether alternative mechanisms also play a role, including brain-immune system interplay, and whether neurological mechanisms alter respiratory control in very ill patients need to be determined. Also unclear is whether different mechanisms predominate at different ages (2).”

Of recent concern have been the “long COVID-19” symptoms in the nervous system, since “they might not become apparent or maximal for weeks or months (or even longer) after the resolution of the acute infection. Symptoms of long COVID-19 are disproportionately neurological and psychiatric in nature, including headache, malaise, joint and muscle pain, anxiety, depression, altered cognition (“brain fog”), and sleep disturbances. Addressing these long-term sequelae is critical for quality of life in patients who have survived acute COVID-19 and will require allocation of appropriate resources and services. Special clinics are being developed to diagnose and treat both children and adults with long COVID-19 and such clinics will provide essential long-term data regarding prognosis, neurological and beyond (2).”

The articles in this initial survey of the impact of the Covid (Sars-CoV-2) pandemic provide a varied sampling of some of the developmental issues associated with the different facets of the pandemic, from the effects of the lock-down itself on normal children's physical and emotional well-being, to the potential biological influences themselves of infection of the pregnant mother on her child. Another contributor looked at what happened to special needs children when the continuity of their care was interrupted and how service providers at one developmental medical center for the evaluation and treatment of those children solved the vital problem of keeping therapeutic and guidance channels open at a time when the world was closing down on them and “turning off the lights” (2).

This is an initial survey. With time, the more intermediate and long- term effects of different aspects of the pandemic will become more apparent to both parents, to educators, mental health professionals, educational and child development therapists, as well as neurologists and other pediatric sub-specialists. It is anticipated that further research on the subject will continue to clarify the initial points made in this special themed issue of the Journal.

## The importance of follow-up

The care of children with developmental disability (DD) was especially challenged during the repeated quarantines during the pandemic. These children required repeated visits and follow-up at developmental centers or *via* visually documented reports and the use of parentally recorded smartphone reports, which were hard to accomplish, given the technical limitations on accessing the treatment centers in many areas. Good care and monitoring using repeated measures are mandated in the care of children at developmental risk during the first five years of life (3). The use of digital technology and communication with developmental care professionals became increasingly critical for many children as time wore on.

## Continuity of care

Continuity of care of children with DD was challenged during the pandemic, because these children require continuity of care. The continuity at each developmental stage helps ensure that the diverse programs and agencies affecting children provide coordinated services. Needed is coordination and communication among practitioners in different fields and systems, since there are diverse types of practitioners and programs within the health care and education sectors. For example, if a toddler is enrolled in an early education center, followed at a DD center and also is receiving early intervention services at home, communication with parents and between the caregivers is critical. The purpose is not only to coordinate services for individual children and their families, but also to create shared understanding of the interconnected quality of developmental processes that each practitioner may see only in part. One result of such continuity is greater opportunities for successful and effective consultation and referrals across professional sectors (4).

## Early intervention

The pandemic and its quarantines presented a unique challenge to the principle of the need for early intervention. The early care of disabled or at-risk children, such as premature babies with an adverse post-natal course, could not wait the pandemic out. Early intervention for children at risk and for those with established intellectual disabilities is now firmly embedded in the context of general early childhood development. An overarching developmental framework has been advanced and has achieved a high level of consensus; one that is relevant to typically developing children and to those vulnerable to a range of developmental problems, particularly intellectual disability. The importance of the convergence of the developmental science of normative development, the developmental science of risk and disability, and intervention science cannot be overstated (5, 6).

## Strengthening the role of parents

The articles in this issue emphasize the role of families as both partners and actual case managers in the treatment and monitoring of their children during the pandemic. Parents and families have the strongest influence on the growth and development of their children, and the aim of family engagement is to bring staff and families together around the common cause of supporting children's development and learning. Families have valuable knowledge to share with the people helping to care for and educate their children, from the characteristics of a particular child to more general cultural funds of knowledge. Educators, administrators (and health care professionals) benefit from taking an approach of respectful inquiry when it comes to understanding families' cultural beliefs and practices around, for example, such issues as eating and sleeping, attachment and separation, and the role of play in learning. As such, it was also important to support parents and families in their understanding of child development in order to engage them in their children's education (and treatment). Care and education professionals promote responsive and culturally appropriate parenting, as well as respect for and understanding of the home language and culture, and encourage or facilitate formal and informal support networks. Moreover, supports provided affect various aspects of life for parents and families, including economic stability, education, and health (7).

## COVID-19 and neuro-development of children

In reviewing the literature on potential neurological effects on the unborn child, great prominence was given to the effects on the pregnant mother. Neurological symptoms among pregnant women with COVID-19 are rare. In a review of the literature (8) it was noted that there were 18 case reports of pregnant women with both COVID-19 and a neurologic complication published until November 2021. The central nervous system and the peripheral nervous system were equally affected, but acute respiratory distress syndrome due to COVID-19 and ICU admission were more frequent among women with central nervous system conditions. Only one case presented a poor neurologic outcome. Both the central and peripheral nervous systems were equally affected: delirium (*n* = 1), posterior reversible encephalopathy syndrome (*n* = 4), cerebrovascular disease (*n* = 2), acute cerebral demyelinating disease (*n* = 1), acute necrotizing encephalopathy (*n* = 1), Guillain–Barré syndrome (*n* = 5), including one patient who also had vestibular neuritis, Bell's palsy (*n* = 3), and rhabdomyolysis (*n* = 1). The median maternal age was 32.5 (25–35) years, the median gestational age was 34 (30–36.5) weeks, and 38.9% presented previous medical conditions. Respiratory symptoms were reported in 76.5%, and 76.5% received immunotherapies to treat the COVID-19 or the neurologic complications (8).

It is not yet clear whether there is a direct effect on fetal brain development *via* vertical transmission of the virus itself. Evidence in the matter is still speculative.

One early series (9) concluded that “even if children are less susceptible to disease complications with mainly mild symptoms, pediatricians are not fully aware of the possible long-term effects of inflammation and/or preterm delivery on brain development.” In their review, they suggested that MIA (maternal immune activation) or a cytokine storm might have an impact on fetal brain development. They therefore concluded that “close monitoring and early intervention in young children born to infected mothers would be highly recommended, especially in the case of preterm babies” (9).

One study stated (2) that in an up-to-date exhaustive review of the literature, no clear cut evidence yet existed of viral effects on the developing fetal brain, making the following points:

1. Vertical transmission of severe acute respiratory syndrome coronavirus 2 (SARS-CoV-2) from mother to fetus is extremely rare.
2. Similar but milder neurological dysfunction occurs in infants and children compared with adults.
3. There is no definitive evidence that SARS-CoV-2 invades the central nervous system.
4. Long-term neurological and psychiatric effects of coronavirus disease are emerging.
5. No data are yet available on variant-specific neurological effects of SARS-CoV-2.

However, evidence is accumulating that SARS-CoV-2 can have post-natal, childhood and later neurological involvement, as well as “long-covid” symptoms and deleterious effects.

## COVID-19 and ASD

Any discussion of COVID-19 and disabilities in children would be remiss in not mentioning the special importance of the effect of social isolation on a subgroup of children, already at a disadvantage regarding their communication and social skills, namely children with autism spectrum disorder (ASD). ASD already figures a prominent position in the allocation of resources of time and manpower at Child Development Centers at medical centers and in the community. Over the past two decades, there has been an increase in measured autism prevalence globally, reflecting the combined effects of multiple factors including the increase in community awareness and public health response globally, progress in case identification and definition, and an increase in community capacity. Approximately 1/100 children are diagnosed with autism spectrum disorder around the world. Prevalence estimates increased over time and varied greatly within and across sociodemographic groups. These findings reflect changes in the definition of autism and differences in the methodology and contexts of prevalence studies (10).

Autism spectrum disorder (ASD) is estimated to affect up to 3% of children in the United States. Public health surveillance for ASD among children aged 4 years provides information about trends in prevalence, characteristics of children with ASD, and progress made toward decreasing the age of identification of ASD so that evidence-based interventions can begin as early as possible (11).

Indeed, given that anxiety, depression, irritability, boredom, inattention and fear of COVID-19 are predominant new-onset psychological problems in children during the COVID-19 pandemic, children with pre-existing behavioral problems like autism and attention deficit hyperactivity disorder have a high probability of worsening of their behavioral symptoms (12).

In one report (13), the majority of parents of ASD children reported a negative impact in emotion management against those in control group reporting mostly positive or no impact. Caregivers reported higher mean scores of anxiety levels in themselves than in their children. ASD children and their parents had higher levels of anxiety than healthy ones. In the group with ASD, children that did not maintain routines had higher mean levels of anxiety than children that maintained routines (13).

Distance learning increased educational deprivation and social inequalities, especially for the youngest children, who lost almost one year of school. The situation was even worse for children with disabilities, who were neglected by the institutions (14). Therefore, a group of researchers in Italy concluded that there was a potential important psychological impact of the COVID-19 pandemic not only in children with neurodevelopmental disorders but in their caregivers as well. As such, physicians must be prepared for the post-pandemic surveillance of mental disorders among families.

## How COVID-19 affects services delivery

In the fall of 2020, close to almost a year after the start of the pandemic, the AUCD (Association of University Centers on Disabilities) and the CDC conducted a rapid needs assessment of disabled children in the United States. The primary purpose of the assessment was to describe the impact of the COVID-19 pandemic on early identification of children with developmental delays and disabilities. The needs assessment identified current and emerging needs, barriers, strengths, and opportunities for early recognition of developmental delay and disability among children from birth to age 5 years during the pandemic. A summary of their key findings was as follows (15):

◾ A majority (91%; *n* = 345) of respondents indicated that the COVID-19 pandemic “highly impacted” early identification of developmental delays and disabilities in young children from birth to age 5 years.
◾ Nearly half (48%; *n* = 131) of respondents reported the number of children served by early childhood programs and systems overall has decreased since the COVID-19 pandemic started. Qualitative data collected from programs and systems indicate that the decrease in children served could lead to a negative impact on early identification.
◾ A majority of programs/systems have transitioned to hybrid service delivery (i.e., a combination of virtual and in-person) since the start of the pandemic, including: 57% of programs/systems that provide referral for early intervention services and 66% of programs/systems that provide developmental and autism screening and early intervention services.

The COVID-19 pandemic has had a multilevel impact on early identification and service delivery including: reduced allocation of resources for this work (e.g., staffing, funding, and time), poor service delivery coordination, communication challenges between families and providers, misconceptions about service availability, competing priorities as families struggle to meet basic needs of living (15).

## Conclusions

In conclusion, the COVID-19 pandemic appears to be waning, but the disabilities research here is just beginning. Our communities mourn the loved ones lost and injured by the virus and the missed opportunities for diagnosis and care of the disabled. However, the lessons learned around the globe point to potential benefits from improved methods of communication and tracking of children and their families, new partnerships in caring and treatment, and better appreciation of the need to act quickly and sensitively when new health challenges appear on the horizon.
